# *Streptomyces* sp metabolite(s) promotes Bax mediated intrinsic apoptosis and autophagy involving inhibition of mTOR pathway in cervical cancer cell lines

**DOI:** 10.1038/s41598-018-21249-5

**Published:** 2018-02-12

**Authors:** Vipin Mohan Dan, Balaji Muralikrishnan, Rahul Sanawar, Vinodh J. S., Bhushan Bapusaheb Burkul, Kalanghad Puthankalam Srinivas, Asha Lekshmi, N. S. Pradeep, Syed G. Dastager, B. Santhakumari, Thankayyan R. Santhoshkumar, R. Ajay Kumar, Madhavan Radhakrishna Pillai

**Affiliations:** 1 Rajiv Gandhi Centre for Biotechnology (RGCB), Thycaud Post, Poojappura, Thiruvananthapuram, Kerala India; 2 NCIM Resource centre, Division of Biochemical Sciences, CSIR - National Chemical Laboratory, Pune, Maharashtra India; 3 Jawaharlal Nehru Tropical Botanical Garden and Research Institute, Palode, Thiruvananthapuram, Kerala India; 4 Proteomics facility, CSIR - National Chemical Laboratory, Pune, Maharashtra India

## Abstract

In cervical cancer, the association between HPV infection and dysregulation of phosphoinositide 3-kinase (PI3K)/protein kinase B (AKT)/mammalian target of rapamycin (mTOR) pathway (PI3K/AKT/mTOR pathway) places mTOR as an attractive therapeutic target. The failure of current treatment modalities in advanced stages of this cancer and drawbacks of already available mTOR inhibitors demand for novel drug candidates. In the present study we identified the presence of a mTOR inhibitor in an active fraction of the ethyl acetate extract of *Streptomyces* sp OA293. The metabolites(s) in the active fraction completely inhibited mTORC1 and thereby suppressed activation of both of its downstream targets, 4E-BP1 and P70S6k, in cervical cancer cells. In addition, it also stalled Akt activation via inhibition of mTORC2. The mechanism of mTOR inhibition detailed in our study overcomes significant drawbacks of well known mTOR inhibitors such as rapamycin and rapalogs. The active fraction induced autophagy and Bax mediated apoptosis suggesting that mTOR inhibition resulted in programmed cell death of cancer cells. The molecular weight determination of the components in active fraction confirmed the absence of any previously known natural mTOR inhibitor. This is the first report of complete mTOR complex inhibition by a product derived from microbial source.

## Introduction

Cervical cancer is the third most common cancer occurring globally among women. GLOBOCAN (IARC) estimates that 527,600 new cases of cervical cancer were reported globally in 2012 with a mortality of 265,700^1^. India shared 25% of the global total, and cervical cancer continues to be the most common cancer in Indian women. Although clinical management of cervical cancer has improved with combination therapies, advance stages of this disease still has very poor prognosis^2,3^. In cervical cancer cases failure of traditional chemotherapy leaves the patient with no other effective treatment options. At the same time while prophylactic vaccines are available in the market they have no role in treating established infections. Development of targeted therapy that focuses on specific molecular pathways deregulated in cancer is therefore important. Alterations in the PI3K/AKT/mTOR pathway are correlated to poor response to treatment in cervical cancer and other solid tumors^4,5^. Human papillomavirus (HPV) is the principal etiological agent for cervical cancer and over 95% of this cancer is positive for oncogenic HPV DNA^6,7^. Persistent HPV infection is known to modulate the network of multiple signaling pathways and in HPV associated cervical cancer PI3K/mTOR/Akt pathway is often derailed^8^. These alterations in the PI3K/AKT/mTOR pathway can be considered ideal targets for the development of suitable drug targets in cervical cancer treatment^9^. This scenario of requirements of newer drugs bring researchers back to nature, given the structural diversity of natural compounds, novel drugs with more specificity, efficiency and safety is an exciting possibility^10^. Microbial based compounds share majority of the drugs in use for human diseases, among which 45% is contribution by the genera actinomycetes^11,12^. Rapamycin the first known mTOR inhibitor, isolated from *Streptomyces hygroscopicus*^13,14^, brought into light a previously unknown molecular target, the mTOR protein complex consisting of mTORC1 and mTORC2, each regulating specific cellular functions^15,16^. The delineation of mTOR pathway revealed its outsized prominence in cell growth, proliferation, recognizing environmental cues to anointing nutrients/energy as per the needs of the cell^17^. The vast influences of this pathway in major cellular functions led to correlating its implications to many pathological diseases. As mTOR pathway gained interest in cancer research, it led to development of newer efficient derivatives of rapamycin called rapalogs^18^. Rapamycin and rapalogs even though came to clinical use, suffered various drawbacks like incomplete inhibition of mTORC1 target 4E-BP1, a negative feedback inhibition that lead to increased Akt activation thereby allowing cancer survival and no significant effect on mTORC2 functions^19^. In clinical scenario, diseased tissue samples from colon and breast cancer patients treated with everolimus (rapalog) showed enormous hike in activated Akt levels compared to pretreatment samples^20^. Such high levels of activated Akt that allows cancer cells to evade apoptosis, coupled with incomplete inhibition of 4E-BP1 proves to be a toxic combination that lead to cancer progression in patients thereby limiting use of rapamycin/raplogs in cancer treatment.

The present study thus explored the microbial community associated to Western Ghats region in Thiruvananthapuram district of Kerala. Screening led to the discovery of *Streptomyces* sp OA293 which produced an active principle that induced Bax mediated intrinsic form of apoptosis accompanied with autophagy. Further mTOR pathway inhibition was observed with complete inhibition of both mTOR proteins. The metabolite(s) in the active fraction efficiently inhibited activating phosphorylations of both targets of mTORC1,p70S6k and 4E-BP1, and controlled Akt activation by inhibiting mTORC2 phosphorylation at Ser2481. LC-MS (Liquid chromatography–mass spectrometry) revealed the prominent components in the active fraction with molecular weights that does not match any known natural mTOR inhibitor. Rapamycin being the only successful mTOR inhibitor from this phylum discovered nearly 40 years back, the results indicated the chances of hitting a novel natural mTOR inhibitor molecule from this phylum of bacteria.

## Results

### Phenotypic and Genotypic identification of microbe

**Figure 1:**
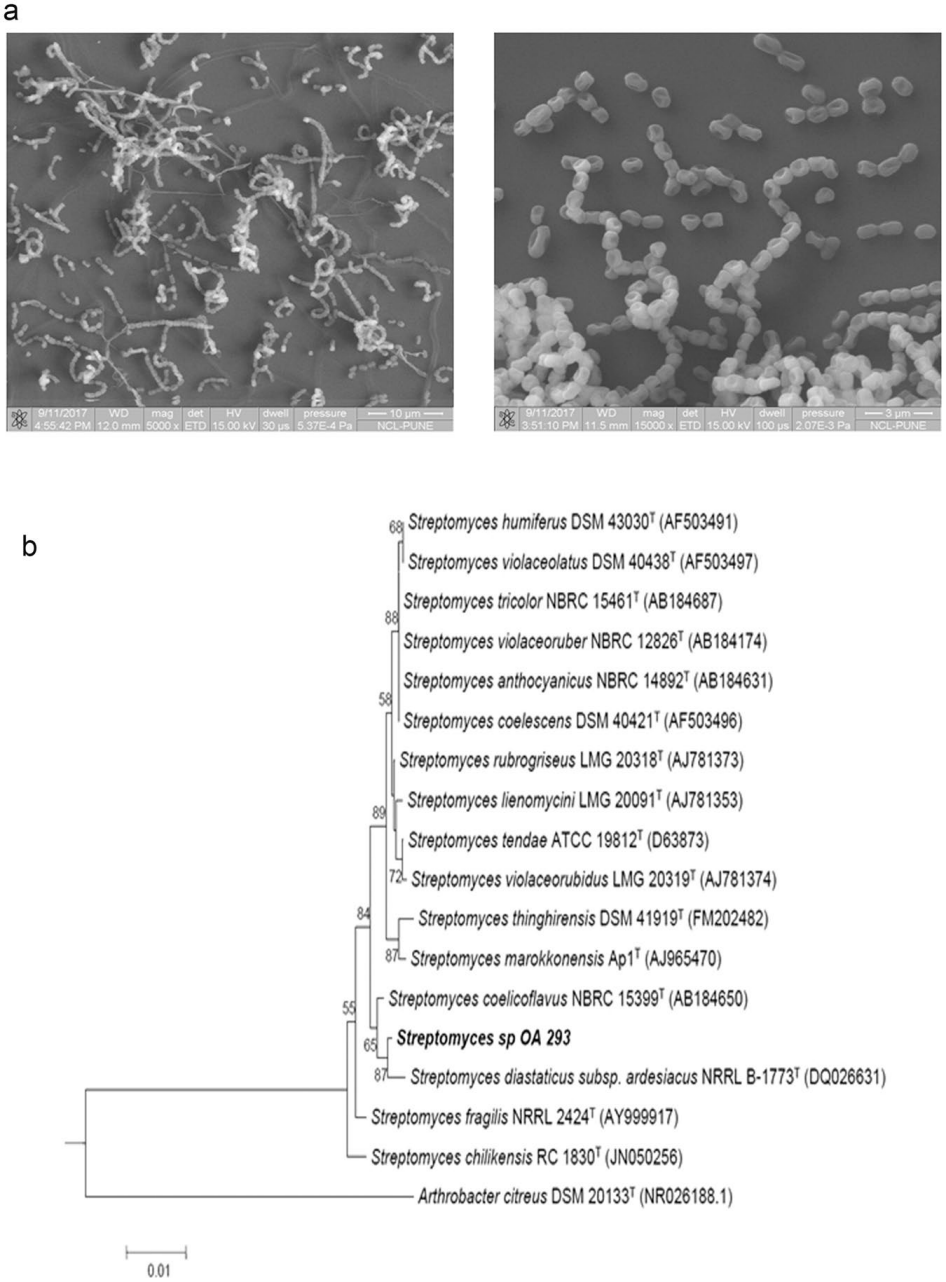
Morphological and phylogenetic characteristics of OA293. (**a**) Scanning electron micrographs indicating the spore chain morphology of *Streptomyces sp* OA 293 at different magnifications. (**b**) Neighbour-joining tree based on 16S rRNA gene sequences showing relationships between *Streptomyces sp* OA 293 and its representatives. Bootstrap values above 50% based on 1000 resampled datasets are shown at branch nodes. Bar represent 0.01 substitutions per site. *Arthrobacter citreus* DSM 20133^T^ was included as outgroup.

Strain OA293 showed proficient growth on ISP 2,ISP 3, ISP 5, ISP6, ISP 7, Bennet’s agar, nutrient agar, Actinomycetes Isolation agar, starch-casein agar after 8–15 days at 28 °C (Supplementary Table S1). The growth was moderate on ISP4 agar. ISP2 was selected to study further characteristics of the bacteria. Gram staining revealed the mycelia structures with long filamentous rods and showed presence of oval shaped spores in chains. The phenotypic, biochemical and morphological features of the strain were evaluated to be determinative to be included in the genus of *Streptomyces* (Fig. 1a, Supplementary Table S2).

16S rRNA sequence of length 1476 bp was obtained. Phylogenetic analysis showcased that *Streptomyces* sp OA293 showed the most similarity to *Streptomyces diastaticus* subsp. ardesiacus NRRL B-1773T and *Streptomyces coelicoflavus* NBRC 15399T as they formed a distinct clade (Fig. 1b). The 16S rRNA gene sequence of strain OA293 was deposited in NCBI/GenBank with Accession ID KY014435.1.

### Determination of fraction with cytotoxic activity

Ethyl acetate extract of OA293 showed significant cytotoxic activity against tested cervical cancer cell lines. The mobile phase constituting chloroform and acetone in the ratio 7:3 gave the most effective separation. The separated fractions obtained in TLC were individually tested for cytotoxic activity. The fraction with the most prominent cytotoxic effect designated SEA4 (Streptomyces Ethyl Acetate fraction 4) was selected for further studies. SEA4 represented a reddish brown component of the extract. SEA4 was dissolved in dimethyl sulphoxide and stored in −20 °C until further use.

### Anti-proliferative activity of SEA4

**Figure 2:**
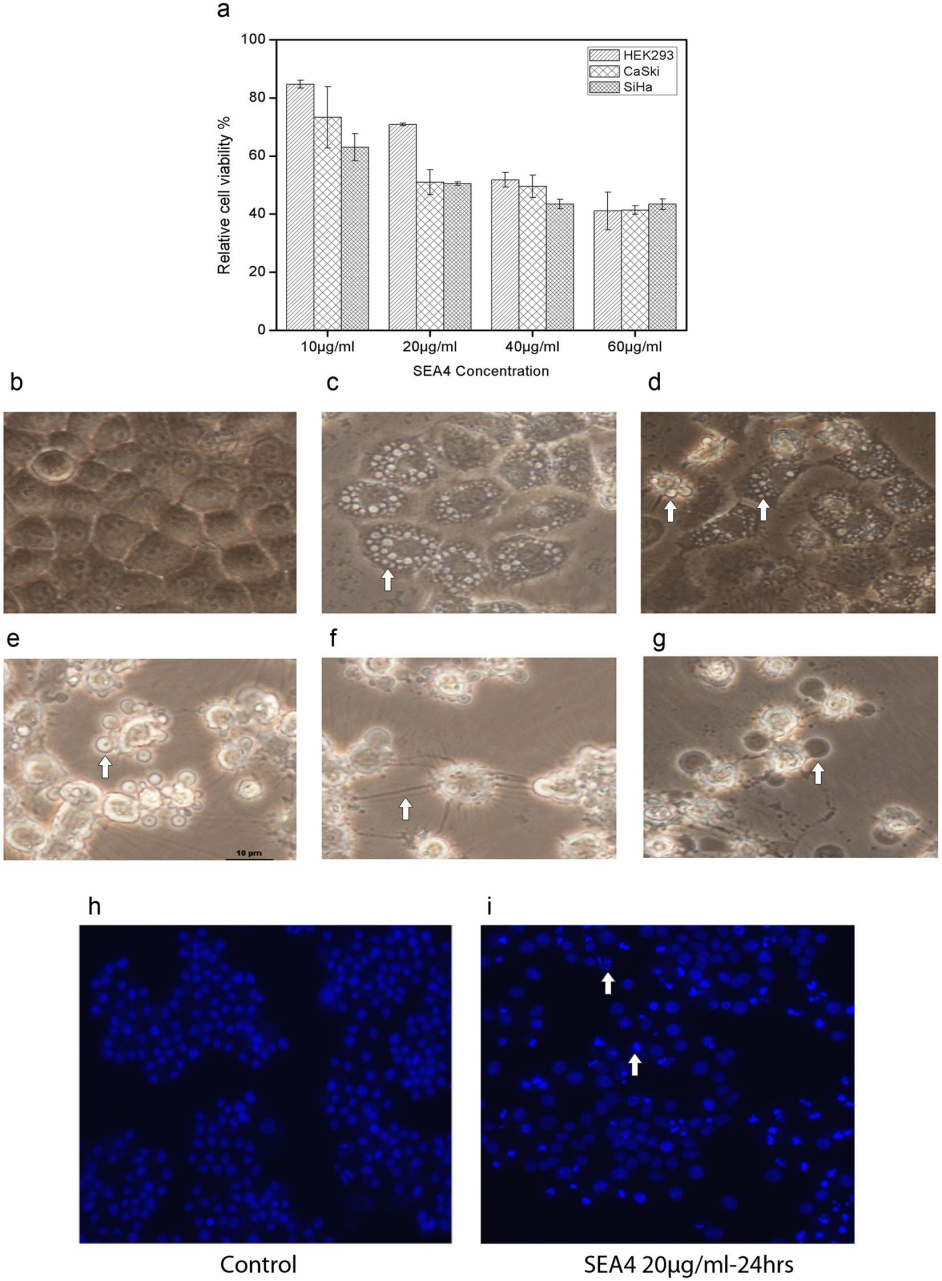
Effect of SEA4 on cervical cancer cell line. (**a**) Antiproliferative activity of SEA4 against cervical cancer cells and normal cell line. MTT assay was employed for assessing the cytotoxic activity. SEA4 was treated at varying concentrations against SiHa, Caski, and HEK(normal cell line). MTT assay for each cell line was carried out as three independent experiments.All data are expressed as mean ± standard deviation. The error bars represent ± S.D. Student’s t test was used for statistical comparison of each cell line to the respective control and difference was considered statistically significant when p ≤ 0.05. All treatments were significant compared to respective control. (**b**) Control SiHa Cell line. (**c**) Presence of autophagic vacuoles in SiHa Cell line at 24 hours of treatment with SEA4 20 µg/mL. (**d**) Figure show cells with autophagic vacuoles and another cell in apoptotic phase at 24 hours (SEA4 20 µg/mL). At 48 hours of treatment characteristics that indicated presence of apoptosis were also noted: blebs (**e**), spikes (**f**), blisters (**g**). Hoechst assay revealed nuclear condensation in treated cells. (**h**) Control Hoechst stained cells. (**i**) Hoechst stained SEA4 (20 µg/mL,24 hours) treated cells with condensed nuclei and arrow mark showing fragmented nuclei.

The anti-proliferative effect of SEA4 was tested against two HPV positive cervical cancer cell lines (Siha and Caski). The concentration of SEA4 at which nearly half population of each cancer cell underwent death was calculated; Siha (20 μg/mL), and Caski (20 μg/mL) (Fig. 2a). In case of normal cell line HEK293 the IC_50_ obtained was at 40 μg/mL (Fig. 2a). All the cell lines employed showed a dose dependant trend of decreasing cell viability with increasing concentration of SEA4.

### Morphological changes induced by microbial extract

Light microscopy observation revealed that the SEA4 induced significant morphological changes in Siha cell line, such as rounding up of cells and detachment from surface of culture plate. The treated cells also showed membrane blebs (Fig. 2e), spikes (Fig. 2f) and blisters (Fig. 2g), which are prominent indications of apoptosis. Certain subsets of the cells showed vacuole formation inside cytoplasm thus indicating possibility of autophagy (Fig. 2c,d). For initial confirmation of apoptosis, nuclear condensation assay was done with Hoechst stain which demonstrated presence of condensed nuclei and formation of apoptotic bodies with fragmented nuclei compared to control (Fig. 2h,i).

### Evaluation of apoptosis induced by SEA4

**Figure 3:**
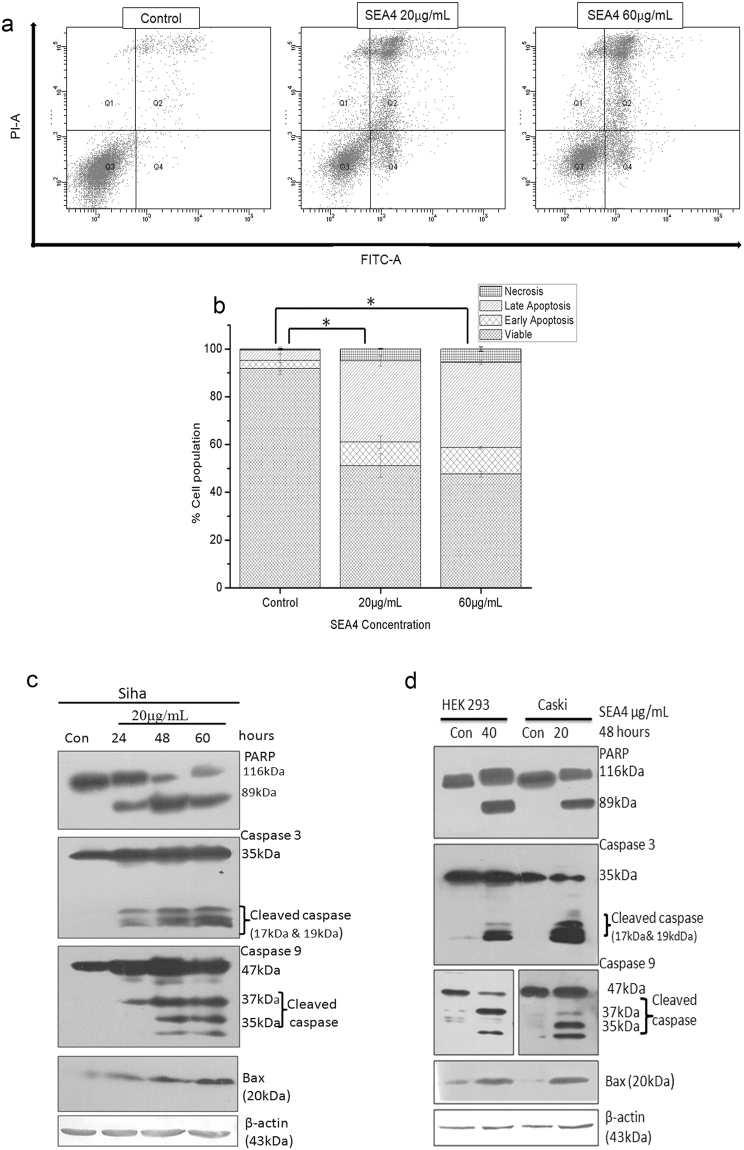
Analysis of programmed cell death induced by SEA4. (**a**) SiHa cells were stained with annexin V-FITC and PI, following treatment with different concentrations SEA4 and was analyzed by flow cytometry. In each panel, lower left quadrant (Q3) shows viable cells which are negative for both annexin V - FITC and PI staining, lower right quadrant (Q4) shows early apoptotic cells which are annexin V positive, upper left quadrant (Q1) shows PI positive cells which are dead/necrotic and upper right quadrant (Q2) shows late apoptotic cells that are both annexin V and PI stained. (**b**) Graphical representation of annexin V-FITC and PI treatment of three independent experiments. All data are expressed as mean ± standard deviation. The error bars represent ± S.D. Student’s t test was used for statistical comparison of control to the two treatments (SEA4: 20 μg/mL, 60 μg/mL) and difference was considered statistically significant when p ≤ 0.05 (*). All treatments were significant compared to respective control. (**c**) Representative of western blots, SEA4 (20 μg/mL) induced cleavage of PARP, activation of caspase enzymes and Bax accumulation there by indicating intrinsic mode of apoptosis in time dependant manner. (**d**) Analysis of status of apoptotic proteins upon treatment with SEA4 in HEK293(40 μg/mL) and caski (20 μg/mL) at 48 hours. Full-length blots are presented in Supplementary Figure 1. Membranes were cut to enable blotting for multiple antibodies.

Presence of apoptotic like features in SEA4 treatment group was investigated further employing Annexin V-FITC(fluorescein isothiocyanate)/Propidium iodide(PI) double staining protocol. Stained cells were subjected to flow cytometric analysis to assess the percentage of cells undergoing apoptosis in a given treatment in contrast to untreated control cells. The analysis relies on the presence of phosphatidylserine that gets translocated to outer membrane during apoptosis, which is eventually recognized by Annexin V-FITC. The scatter plot of control and treated samples demonstrating four distinct population of unstained viable cells, Annexin V stained early apoptotic cells, PI stained necrotic cells and PI/Annexin dual stained late apoptotic cells is shown in Fig. 3a. The SEA4 treatment witnessed significant accumulation of cells in the early apoptotic and late apoptotic phase thus denoting significant apoptosis in treatment groups compared to control (Fig. 3a,b).

To further confirm the nature of apoptosis, expression pattern of key apoptotic regulators such as PARP, caspase 3, caspase 9 were analyzed by western blotting. PARP cleavage is a prominent marker of apoptosis and signifies activation of caspase enzymes that carry out the programmed cell death process. Efficient cleavage of PARP and activation of caspase 3 and caspase 9, were also observed (Fig. 3c). Bax, known to be a critical early regulator of mitochondrial apoptosis was anlayzed in treated samples. As shown in Fig. 3c a significant upregulation of Bax was observed in SiHa from 24 hours, confirming Bax regulated mitochondrial permeabilization. We also analyzed the molecular events of apoptosis in caski and HEK 293 at their respective IC_50_ concentration (Fig. 3d).

**Figure 4:**
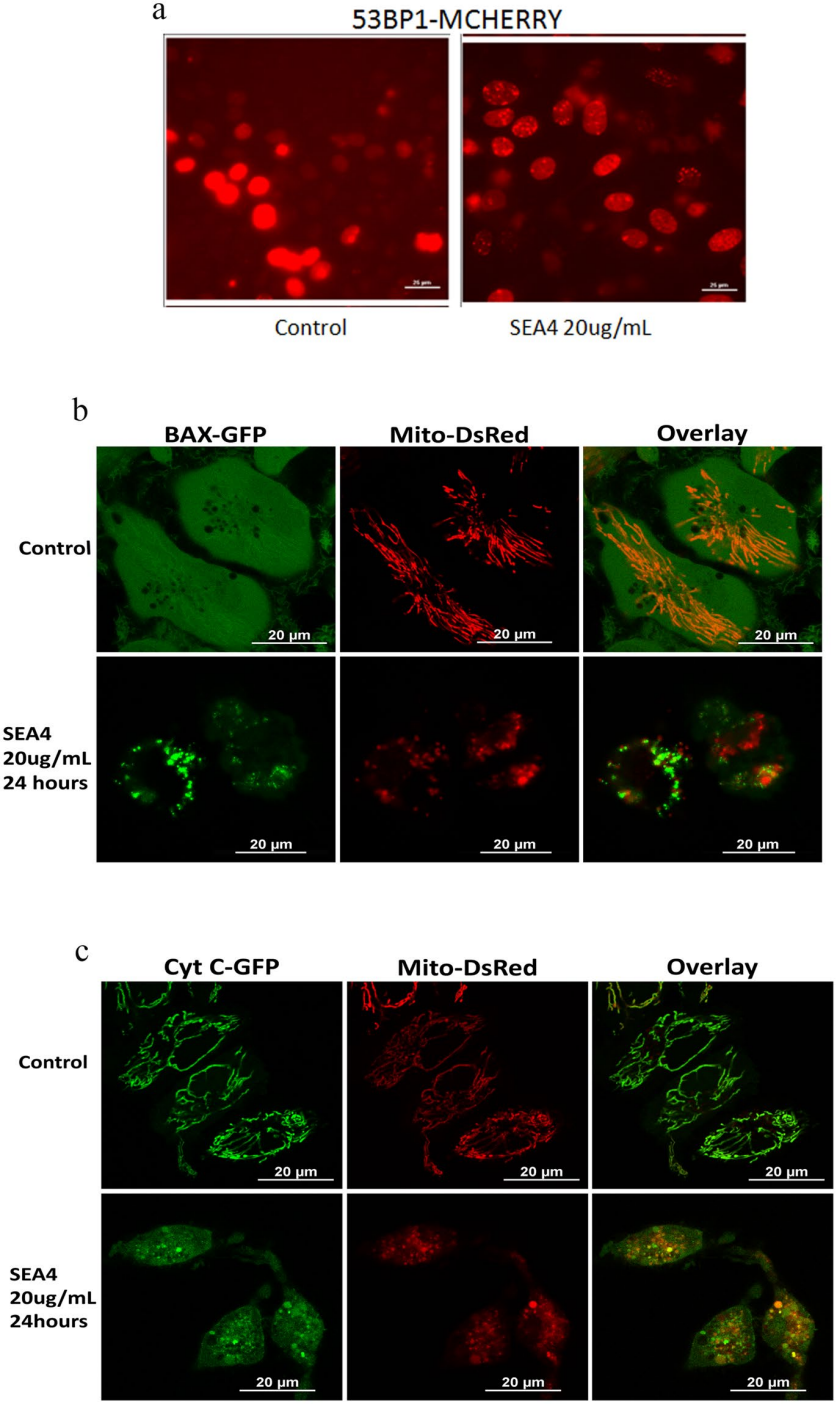
Confocal imaging of apoptosis related proteins in response toSEA4. (**a**) SEA4 (20 μg/mL, 24 hours) treatment witnessed formation of focal points signifying DNA damage and recruitment of 53bp1 to site of damage. (**b**) Confocal imaging of Bax EGFP-Mito-DsRed cells substantiated translocation for Bax to mitochondria induced by SEA4 treatment. (**c**) The release of cytochrome c from mitochondria in treated cells expressing cytochrome EGFP and Mito DsRed confirmed intrinsic mode of apoptosis induced by SEA4.

**Figure 5:**
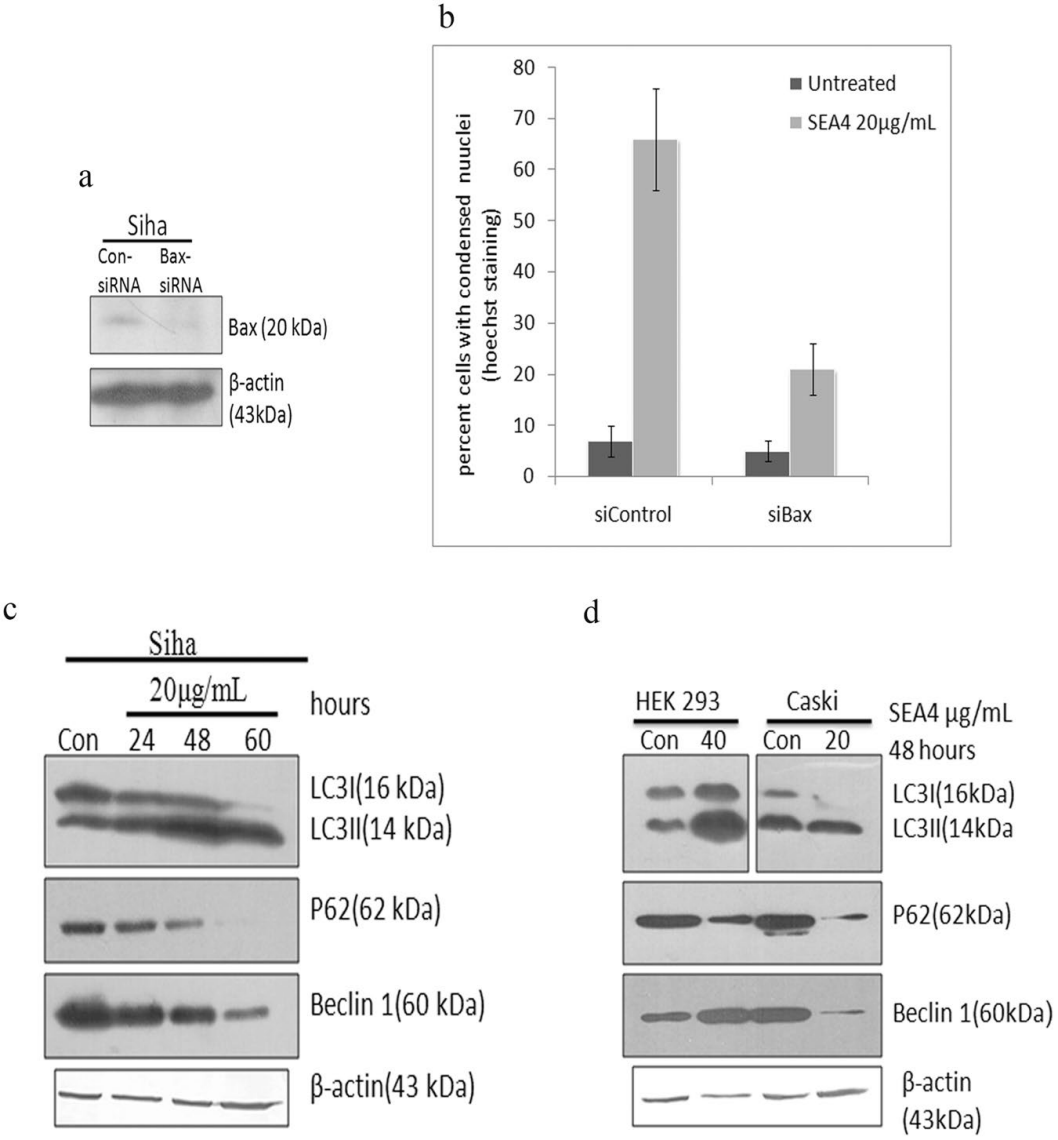
SEA4 induced Bax mediated apoptosis and also induced autophagy in cervical cancer cells. (**a**) Bax was silenced in Siha Cells leading to knockdown of Bax. (**b**) Silencing of Bax reduced cell death from 66% in control siRNA silenced -SEA4 (20 μg/mL/24 hours) treated Siha cells to 21% in Bax siRNA silenced-SEA4(20 μg/mL/24 hours) treated Siha cells. (**c**) Representative of western blot, SEA4 (20 μg/mL) induced beclin 1 reduction and LC3I to LC3II conversion progressively in a time dependent manner. Similarly p62 showed decreasing pattern of expression with time there by confirming autophagy. (**d**) Induction of Autophagy by SEA4 was also observed Caski and HEK293. Reduction in Beclin 1 was observed in caski cells upon treatment with SEA4.In HEK293 Beclin 1 showed an increase in accumulation. Full-length blots are presented in Supplementary Figure 1. Membranes were cut to enable blotting for multiple antibodies.

53BP1 (p53-binding protein 1) is an intergral part of the DNA-damage response (DDR) signal transduction cascade that responds to gentoxic insults. In the event of DNA damage this mediator protein undergoes nuclear localization and oligomerization thus binding to site of DNA damage forming focal structures^21^. SEA4 treatment lead to DNA damage thus recruiting 53BP1 to the site of damage. The formation of focal points were observed in our confocal images (Fig. 4a). These focal points serves as platform for binding of other DDR proteins which signals various other effector molecules thus leading to appropriate biological response, including apoptosis^21^. Cytochrome c being a crucial part of apoptosis we analyzed the release of this protein from mitochondria using cells expressing cytochrome EGFP and Mito DsRed. Cytochrome c release was witnessed in our study upon treatment with SEA4 indicating their loss from Mito-DsRed (Fig. 4c). In addition, confocal imaging of Bax EGFP-Mito-DsRed cells substantiated translocation of Bax to mitochondria (Fig. 4b). To confirm Bax mediated apoptosis induced by SEA4, Bax was silenced in SiHa Cells (Fig. 5a). As shown in Fig. 5b silencing of Bax reduced cell death from 66% in control siRNA silenced, SEA4 (20 μg/mL) treated SiHa cells to 21% in Bax siRNA silenced-SEA4 treated Siha cells suggesting essential role of Bax in mediating apoptosis. Overall the study supports Bax mediated intrinsic mode of apoptosis by SEA4.

### Induction of Autophagy by SEA4

Microscopic analysis revealed the presence of vacuole formation (Fig. 2c) in certain subset of SEA4 treated cells and hence further evaluation of autophagic related proteins was done. Autophagy involves the conversion of cytosolic form of LC3I to lipidized autophagosome associated form LC3II. In treated Siha cells, the active extract progressively induced the LC3I to LC3II conversion and also reduced the levels of p62(SQSTM1) thus ensuring the formation of autophagasome (Fig. 5c). To further confirm the progression of autophagy the status of Beclin1 was analyzed, another prominent marker of autophagy that accumulates upon of initiation of autophagy. Interestingly Beclin 1 showed a progressive reduction with SEA4 treatment with respect to increasing incubation period of SEA4 (Fig. 5c). Induction of Autophagy by SEA4 was also observed in Caski (20 μg/mL) and HEK293 (40 μg/mL) (Fig. 5d). Beclin 1 was also reduced in Caski cells upon treatment with SEA4 (Fig. 5d), while in HEK293 Beclin 1 showed an increase in accumulation.

### Status of mTOR complex and its effector proteins

**Figure 6:**
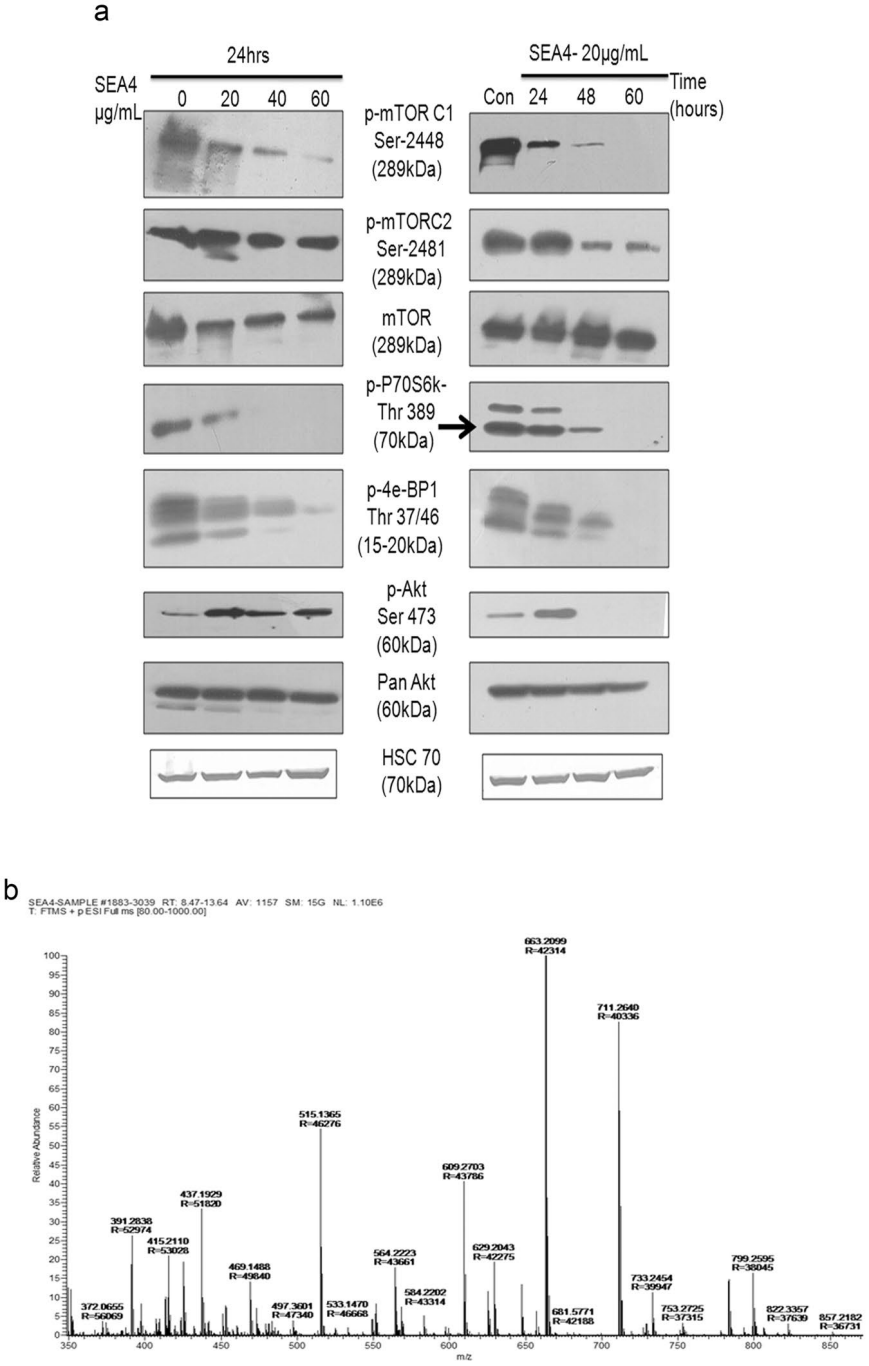
SEA4 treatment leads to inhibition of mTOR pathway and molecular weight analysis of components in SEA4. (**a**) SEA4 showed mTOR pathway inhibition in both time course (24 hours, 48 hours and 60 hours of 20 μg/mL) and concentration dependent manner (20 μg/mL, 40 μg/mL and 60 μg/mL at 24 hours) of treatments. mTORC1 was observed to be the primary target of compound(s) in SEA4, inhibiting specific activating phosphorylations of key target proteins mTORC1 (Ser 2448), 4E-BP1(Thr-37/Thr46) and p70s6K (Thr 389). mTORC2 was inhibited at a later stage thus causing an initial (24 hours) hike in activated Akt (Ser473), further time points (48 hours and 60 hours) witnessed a significant reduction in activating phosphorylations of both mTORC2 (Ser2481) and Akt (Ser 473). Phospho-Akt (Ser 473) blots are obtained after longer duration of exposure. Thus SEA4 was able to effectively inhibit both components of the mTOR complex. (**b**) Mass spectrum of SEA4 showing molecular weight of components. Full-length blots are presented in Supplementary Figure 1. Membranes were cut to enable blotting for multiple antibodies.

As SEA4 was able to induce autophagy and apoptosis in cancer cells, we investigated the status of mTOR (mechanistic target of Rapamycin), a serine/threonine kinase known to be a major effector of cell growth and proliferation. mTORC1 upon activation phosphorylates and activates two proteins, 4E-BP1 and S6K, these proteins then progress to aid in translation, lipid synthesis and various cellular functions^22^. mTORC2 instruments various cellular function by phosphorylating Akt specifically at Ser473^22^. The activation of mTOR C1 and mTORC2 can be confirmed by the status of their phosphorylation sites, Ser 2448 and Ser 2481, respectively, and also by determining the status of phosphorylation status of downstream proteins; p70S6K at Thr-389/4E-BP1 at Thr37/46 and Akt at Ser-473. mTORC1 showed a rapid decline in phosphorylation at Ser 2448 with increasing time period as well as increasing concentration of SEA4 treatment in SiHa (Fig. 6a). The inhibition pattern of mTORC1 (Ser 2448) by SEA4 was in correlation to the reduced phosphorylation of its downstream proteins P70S6K1 and 4E-BP1 (Fig. 6a). The inhibition of all the three above proteins was observed in a progressive manner in regard to time scale (20ug/mL for 24 hour, 48 hour, 72 hour) and concentration scale (20 ug/mL, 40 ug/mL, 60 ug/mL for 24 hours) in SiHa (Fig. 6a).The highest concentration as well as the highest time point witnessed complete abolition of phosphorylation of the target proteins studied, thus showcasing effective inhibition of mTORC1 (Fig. 6a). In regard to mTORC2, at 24hrs of incubation with SEA4, we observed a hike in phosphorylation of Akt at Ser473 and with mTORC2 retaining phoshorylated (Ser2481) active state. The time scale points of 48 hours and 60 hours showed effective reduction in phosphorylation in both mTORC2(Ser 2481) and Akt (Ser 473) (Fig. 6a). This suggested inhibition of mTORC2 was delayed in the mechanism employed with the primary target being mTORC1,but however later mTORC2 succumbed to significant downregulation in its activity. The results obtained thus substantiated effective inhibition of mTOR complex.

### LC-MS anlaysis of SEA4

SEA4 was analyzed and based on respective relative abundance of components the mass spectrum was obtained (Fig. 6b). None of the components in SEA4 fraction were closer to molecular weight of any known natural mTOR inhibitor, thereby ensuring presence of a new natural mTOR inhibitor.

## Discussion

The clinical management of cervical cancer heavily depends on target based anticancer therapy using, erlotinib and bevacizumab. Even though these are promising therapies they show limited efficiency^23^. The failure or less effectiveness of current therapies can be owed to various factors ranging from clonal/genetic heterogeneity to cell signal complexity of cervical cancer^2,23^. Considering the reported alternations in PI3K/mTOR/Akt pathway in cervical cancer, mTOR inhibitors may play a vital role in treatment.

In the current study, we describe the mechanism of action of a mTOR inhibitor in an active fraction of the ethyl acetate extract of *Streptomyces* sp OA293. Treatment with SEA4 induced two major events in the treated cancer cell line, apoptosis and autophagy. Earlier reports suggest that compounds such as carnasol, vitamin K and resveratrol are known to induce both these events upon treatment^24–26^. Apoptosis is a major cell death mechanism utilized by many chemotherapeutic agents targeting the cancerous cell to a silently programmed self suicide^27,28^. The activation of various apoptosis related proteins revealed the intrinsic mode of cell death activated by the extract fraction, SEA4. Since the active fraction induced significant upregulation of Bax protein in the cell line, it appears that Bax dependant mitochondrial permeabilization plays as an initiator of intrinsic pathway of apoptosis that culminates in caspase activation and PARP cleavage.

Autophagy is a cellular degradation mechanism that helps in channeling of unwanted proteins or damaged organelles to lysosomal degradation. Autophagy contributes to homeostasis by functioning at basal level and is taken to higher degree of action when the cell undergoes variety of cellular stress conditions. SEA4 induced autophagy in SiHa is through a mechanism involving downregulation of Beclin1. Western blot analysis revealed a significant progressive reduction of Beclin 1 with increasing incubation period of SEA4. These progressive reductions led us to believe that Beclin 1 is probably targeted for cleavage by activated caspases. A cellular model system employed by Wirawan and coworkers (2010)^29^ explained that beclin 1 can be targeted by activated caspases at specific cleavage points on this autophagic protein. Bax overexpression is also known to trigger the intrinsic mode of apoptosis eventually leading to cleavage of beclin 1 at position D149 by activated caspases^30^. Many recent researches have also supported that beclin 1 independent autophagy augments apoptosis^31,32^. These observations prompted us to check for possible presence of caspase cleaved products of Beclin 1 of molecular weight 37 and 35 kDa. Repeated western blot analysis showed no presence of cleaved products thereby making us to conclude that decreased expression of Beclin 1 is possibly a downregulation at transcriptional/translational level and not resultant of caspase action. Further studies are required to warrant a conclusion.

As the results obtained suggested the presence of an inhibitor of mTOR pathway in SEA4, we searched for other reported mTOR inhibitors from phylum actinobacteria. Rapamycin is the first reported natural mTOR inhibitor from this phylum and Anthracimycin being the latest inclusion of mTOR inhibitor from actinobacteria^14,33^. Rapamycin is in clinical use as an immunosuppresent and its analogue, Temsirolimus was approved by FDA in 2007 for treatment of renal cell carcinoma^14,34^. The mechanism of action of SEA4 was evidently different from these two compounds thereby suggesting the possibility of a novel mTOR inhibitor.

The mTOR complex being combination of two proteins, mTORC1 and mTORC2, determining which among these is affected by SEA4 is important to understand the mechanism of cell death induction. The inhibition in phosphorylation of P70S6K at Thr 389, a direct site of activation by mTORC1, established that mTORC1 kinase activity was progressively abrogated by SEA4. In case of 4E-BP1, a complex series of phosphorylation events at specific sites (Thr-37, Thr-46, Ser-65, and Thr-70) on the protein determines its binding and inhibiting capacity to eIF-4E, thereby regulating progress into translation^35,36^. Thr37/46 on 4E-BP1 is directly activated by mTORC1 and this phosphorylation act as a priming event that initiate phosphorylation of Ser-65 and Thr-70^35^. Thr37/46 phosphorylation event is rapamycin insensitive^37^. In our study SEA4 effectively inhibited phosphorylation on this site, thus indicating the possibility of a different mechanism than that of Rapamycin employed by the compound(s) in SEA4.When an activated mTORC1 phosphorylates 4E-BP1 at Thr-37/Thr-46, the event introduces a conformational change in 4E-BP1 that exposes the other moieties (Ser-65 and Thr-70) which is phosphorylated by other kinases^35^. This array of phosphorylation events on 4E-BP1 promotes its dissociation from eIf-4E, which further proceeds to form the eIf-4F complex that lead to translation initiation^35,38^. Rapamycin is capable of only inhibiting the phosphorylation of Ser 65^37^, but this inhibition does not have significant effect on the functional activities of 4E-BP1. It can be postulated that as SEA4 treatment lead to significant inhibition of phosphorylation at Thr-37/Thr46, thus the conformational changes in 4E-BP1 failed to occur leading to unavailability of other moieties in this protein for further phosphorylation, thus confirming an effective inhibition of 4E-BP1 than availed by rapamycin and rapalogs. In rapamycin/rapalogue treatment, even though p70S6k remains inhibited throughout, incomplete inhibition of 4E-BP1 leads to sufficient activation of translational machinery that supports proliferation of cancer cells^20^. Thus in SEA4 treatment effective inhibition of both P70S6k (Thr 389) and 4E-BP1(Thr37/46) consolidates complete inhibition of mTORC1 thereby halting all related downstream functions.

As part of the growth promoting feature of mTORC1/S6k signal axis, an accompanied negative feedback loop is operated through which signalling events through insulin/IGF (Insulin Growth Factor) receptor and various tyrosine kinase receptors is halted via phosphorylation and transcriptional inactivation of IRS-1(Insulin Response Element)^39,40^. In the event of mTORC1 inhibition by rapamycin/rapalogs this negative feedback loop is broken resulting in IRS-1 activation leading to PI3k/Akt activation in a range of cancer cell lines^41^. This results in high levels of activated Akt in rapamycin/rapalogs treated cancer cells leading to activation of survival pathways thus nullifying anticancer effects of the drug. Owing to the inability of rapamycin/rapalogs to inhibit mTORC2, the activation of Akt goes unchecked leading to cancer survival. In our treatment at a later stage SEA4 treatment showed inhibition of phosphorylation at Ser2481 on mTORC2 leading to its inactivation thus controlling further Akt activation. At the initial stage as witnessed at 24hrs the inhibition of mTORC1/S6k signal and failure in inhibition of mTORC2 must have led to upregulation of phosphorylated Akt (Ser473). The further mTORC2 inhibition action could enable the cancer cell to escape from apoptosis inhibitory survival signals activated by phosphorylated Akt and thus leading the cell through programmed cell death.

The study suggests the possibility of finding actinomycetes candidates from geographical areas which were never explored for therapeutic compounds. The study also presents the opportunity of finding new molecule with mTOR inhibition more efficient than rapamycin, thus opening newer dimensions in the area of mTOR inhibitors.

## Materials and Methods

### Materials

Antibodies against PARP, Caspases, Bax, mTOR (Ser2448), mTOR (Ser2481), Pan Akt, p70S6K (Thr-389), 4E-BP1 (Thr37/46), Akt (Ser473), Pan Akt, LC3. Beclin 1 and p62 were purchased from Cell Signaling (Beverly, MA, USA). Vinculin and β- actin were purchased from Santa Cruz Biotechnology (Santa Cruz, CA, USA). Chemicals for extraction, chromatography and LC-MS were purchased from Merck and other chemicals were purchased from Sigma.

### Isolation and maintenance of microbes

Strain OA293 was isolated was isolated from soil samples collected from garden soil located in Mannamoola, Peroorkada municipal corporation (coordinates-8.537250°N 76.96650°E) Trivandrum district of Kerala, India. The soil sample was collected, transported in sterile plastic bags and stored in ambient conditions in the laboratory. Air dried soil samples were then treated at 55 °C for 15 minutes in a fluid suspension containing an osmoprotectant (Ringer’s solution), serially diluted up to 10^−5^ and plated on starch casein agar (SCA) and Bennet’s agar (BA) supplemented with nalidixic acid (25 µg/mL) and secnidazole (25 µg/mL). The plates were incubated for 15 days, and colonies selected based on its morphology. The selected colonies were initially maintained in their respective isolation media and stored at −80 °C in 10% glycerol.

### Morphological, biochemical and physiological characterization

The cultural, morphological and physiological characterization of the strain OA293 was carried out according to the standard protocol of the International Streptomyces Project^42^. Strain OA 293 was assessed for its growth in various media like ISP 2, ISP 3, ISP 4, ISP 5, ISP 6, ISP 7 agar, Bennet’s agar and Starch-casein agar for 14 days at 28 °C. Gram staining of these bacteria was performed as per the protocol of standard gram reaction^43^ and observed using a Leica microscope. The colour of the mycelium (aerial and substrate) and that of the diffusible pigment were recorded after fourteen days of incubation by comparing with colour chips from ISCC–NBS colour charts standard sample no. 2106^44^.

The morphology of the spores was observed by the use of scanning electron microscopy (FEI Quanta 200 3D). The growth at varying temperatures was assessed by incubating the cultures at 15 °C, 28 °C, 37 °C and 50 °C in ISP2 medium. Tolerance against sodium chloride was tested at concentrations ranging from 0% to 5% in ISP2. The pH range for growth was analysed from pH 6 to 10. Acid production from carbohydrates was carried out by methods described by Shirling and Gottlieb^42^. The hydrolysis of specific substrates like Casein, Starch, Gelatin and DNA were carried out as reported by Williams *et al*.^45^.

### Molecular analysis

DNA extraction waMarmur^46^ (1961). Nearly full length 16S rRNA genes were amplified using the universal primers 27F (5′-AGAGTTTGATCCTGGCTCAG-3′) and 1492R (5′-TACGGCTACCTTGTTACGACTT-3′). The PCR conditions consisted of an initial denaturation at 95 °C for 5 min, 35 cycles of 94 °C for 30 s, 55 °C for 1 min and 72 °C for 1 min 30 s and a final elongation at 72 °C for 7 min. PCR amplicons were purified using Exo-SAP (USB) and sequenced for complete 16 S rRNA gene in ABI 3500xl genetic analyzer (Invitrogen/Life Technologies).

The obtained16S rRNA sequence was subjected to a BLAST search using the EzBioCloud server (http://www.ezbiocloud.net/eztaxon)^47^. The first sixteen hits which showed maximum homology with the sequence of OA293 were selected. These sequences were aligned using ClustalW2 and the phylogenetic relationship inferred by MEGA 6 using the neighbour-joining algorithm^48,49^.

### Fermentation and Extraction

The strain was maintained in ISP2(International Streptomyces Production) medium. A seed culture of OA 293 was grown in ISP2 medium at 28 °C for 40 h on a rotary shaker operating at 200 rpm. This seed culture was subsequently transferred into a fresh ISP2 (Glucose 4 g/L, Yeast extract 4 g/L, Malt extract 10 g/L, pH = 7.2) broth with 0.20% calcium carbonate and fermented for eight days at 200 rpm, 28 °C. After fermentation, the cell pellet was removed by centrifugation, with the resulting supernatant being filtered using a Whatman filter paper No4, after which it was lyophilized. This lyophilized powder was extracted using ethyl acetate, a process which lasted 6 hours. This extract was then concentrated using a Rota evaporator to obtain a dark brownish powder. The final concentrate was suspended in DMSO prior to an anticancer activity screening.

### Identification of bioactive fraction

Ethyl acetate extract showed significant anticancer activity. In order to identify the active fraction, crude extract was subjected to thin layer chromatography (TLC) on silica gel-G (0.5 mm thickness) with various combinations of organic solvents to find the appropriate mobile phase for effective separation of components of the extract.

### Cell cultures

The cytotoxic potential of the extract was tested against human cervical cancer cell lines (Siha and Caski) and normal cell line (HEK293). The cell lines used in the study were purchased from ATCC, used within 3–4 passage numbers after reviving from liquid nitrogen frozen samples. All cell lines were maintained in Dulbecco’s Modified Eagle’s Medium (DMEM) supplemented with 10% fetal bovine serum (Pan life sciences, USA) and antibiotics (Streptomycin 0.1 mg/ml).

### Determination of Anti-proliferative effect

The anti-proliferative potential of the microbial extract was determined by protocol established by Edmondson *et al*.^50^ using 3-(4,5-dimethylthazol-2yl)-2,5-diphenyl tetrazolium-bromide (MTT).

### Nuclear condensation assay

Nuclear condensation assay was performed using Hoechst 3342. Cells were seeded into 24 well plates at 10,000 cells per well. After 24 h incubation spent media was aspirated and the cells were treated with different concentrations of the bacterial extract in culture media. After 24 h, each well was incubated with Hoechst 3342 (5 µg/mL) for 15 min at 37 °C in carbon dioxide incubator. The wells were washed twice in PBS and were analyzed through fluorescence microscope (Nikon, Japan) under UV filter sets, image captured and anlayzed using NIS Elements Version 0.410.

### Annexin assay

Cells were collected from both the control and treatment batches by trypsinization after respective incubation periods. The cell suspension was centrifuged for 6 min at 3000 rpm and washed twice with PBS. Annexin V-FITC Early apoptosis detection kit (#6592) was purchased from Cell Signaling Technology (CST). Flow cytometric analysis was performed as per instructions given in kit.

### Confocal microscopy

SiHa cells that were transfected and expressing 53BP1 fused with mCherry (pLPC-Puro plasmid procured from Addgene (#19835)) was used to monitor DNA damage induced by the active fraction. The stable cells were seeded into 96 well plates and incubated for 24 hours at 37 °C with 5% CO_2_. SEA4 was added at a concentration of 20ug/mL, incubated for 36 hours and the cells were imaged. 53BP1-mcherry was imaged using the filter combination of ex: 545 ± 30, em: 620 ± 60 and dichroic 570LP. The plates were imaged using a 40× Plan Apo λ 0.95 NA objective under an inverted fluorescence microscope (Nikon Eclipse, TE2000-E, Tokyo, Japan).

To analyze cytochrome c EGFP release from mitochondria, cells expressing cytochrome c EGFP and MitoDsRed, were grown on 8 well chambered cover glass. After indicated treatment, cells were imaged with NIKON A1R confocal microscope using 488 and 562 nm laser excitation^51^. The images were captured using EMCCD camera Andor iXON and analysed using NIS Elements AR 3.2 software. For the analysis of Bax translocation to mitochondria, cells expressing Bax EGFP and MitoDsRed were imaged as described above.

### Bax silencing and quantification of chromatin condensation

For silencing of Bax,cells grown on 60 mm dishes were transfected with Bax si RNA (sc-29212) or control si RNA (sc-37007) procured from Santacruz Biotechnology using siRNA transfection kit (sc-29528) from Santacruz as per the manufactures protocol. Bax antibody (N-20) sc 493 was used for the detection of Bax protein by westernblot. For quantifying apoptosis using chromatin condensation, cells were stained with Hoechst and number of cells with condensed chromatin were counted to calculate percentage of cells with condensed chromatin.

### Western blot analysis

Total protein was isolated from control and treated cells using RIPA buffer (50 mM Tris-HCL, pH 8.0, with 150 mM sodium chloride, 1.0% Igepal CA-630 (NP-40), 0.5% sodium deoxychlorate, and 0.1% sodium dodecyl sulfate) that had added combination of protease inhibitor (500 mM phenylmethylsulfonyl fluoride). Bradford method was employed to measure protein concentration. Equal amount of supernatant protein (50 μg) from the control and different treatments were denatured by boiling for 6 minutes in SDS sample buffer and then proceeded for SDS-PAGE, transferred to nitrocellulose membranes for immunoblotting. Membranes were blocked with 5% skim milk in Tris-buffered saline with Tween 20(TBST) [10 mM Tris-HCl (pH 7.6), 150 mM NaCl, and 0.5% Tween 20] and probed against specific antibodies for overnight. Then the membrane was washed thrice for ten minutes each in TBST and probed with secondary antibody for 2 hours in room temperature. The membranes were again washed with TBST (Tris buffer saline- Tween20) as before and developed in dark room.

### LC-MS (Liquid chromatography–mass spectrometry)

The LC-MS data were collected on a Q-Exactive Hybrid Quadrupole-Orbitrap Mass Spectrometer (Thermo Scientific) coupled to Accela UHPLC system using Accucore C18 column of dimension, 150 × 2.1 mm, particle size 2.6 μ (Thermo Scientific) for separation of compounds. The sample volume of 1.50 µl was injected with a gradient program comprising of mobile phase system: solvent A (water) and solvent B (Acetonitrile). The linear gradient started with 5% B at 0 min to 95% B in 15 min at a flow rate of 350 μl/min and 30 °C. Mass spectra were acquired in positive electrospray ionization mode ranging with mass range from 100 to 1000 m/z and the data acquisition and processing were performed using Thermo Scientific Xcalibur software (Version 3.0).

### Statistical analysis

All experiments were carried out in triplicates or duplicates. Results were expressed as mean ± standard error of the mean (SEM). Experimental comparisons between treatments and control were made by Student’s t test with statistical significance set at p < 0.05. Graphical representations were made with Originpro8 (Origin lab, USA).

### Data and material availability

The data generated during and/or analysed during the current study are available from the corresponding author. The strain OA293 won’t be currently publicly available as work is ongoing to isolate and characterize the compound of interest, once compound is identified patent filed and granted the strain will be deposited in recognized repositories. The strain will be available from the corresponding author on reasonable request and on agreements stated by the author to protect the ongoing research interests of the author.

## Electronic supplementary material

Supplementary file
