# Supplementary material for: Streptomyces sp metabolite(s) promotes Bax mediated intrinsic apoptosis and autophagy involving inhibition of mTOR pathway in cervical cancer cell lines
Source: Sci Rep. 2018 Feb 12;8:2810. doi: 10.1038/s41598-018-21249-5 (PMC5809390; doi:10.1038/s41598-018-21249-5)
Supplement: Supplementary file 1 — Supplementary file [file 41598_2018_21249_MOESM1_ESM.pdf]

***Streptomyces* sp metabolite(s) promotes Bax mediated intrinsic apoptosis and autophagy involving inhibition of mTOR pathway in cervical cancer cell lines.**

Vipin Mohan Dan[1,3#], Balaji Muralikrishnan[1#], Rahul Sanawar[1#], Vinodh J S[1,2], Bhushan Bapusaheb Burkul[4], Kalanghad Puthankalam Srinivas[1], Asha Lekshmi[1], N S Pradeep[3], Syed G. Dastager[2], B.Santhakumari[4], Thankayyan R.Santhoshkumar[1*], R.Ajay Kumar[1*], Madhavan Radhakrishna Pillai[1]

[1]Rajiv Gandhi Centre for Biotechnology (RGCB), Thycaud Post, Poojappura, Thiruvananthapuram, Kerala, India.

[2]NCIM Resource centre, Division of Biochemical Sciences, CSIR - National Chemical Laboratory, Pune, Maharashtra, India.

[3]Jawaharlal Nehru Tropical Botanical Garden and Research Institute, Palode, Thiruvananthapuram, Kerala, India

[4]Proteomics facility, CSIR - National Chemical Laboratory, Pune, Maharashtra, India.

Supplementary Table S1: **Culture characteristics of *Streptomyces* sp OA293 on various media at 28˚C after 14 days**

Medium	Growth	Diffusible pigment	Aerial mycelium	Substrate mycelium
Yeast Extract Agar (ISP 2)	Excellent	Red	Yellowish Grey	Blackish Red
Oatmeal agar (ISP 3)	Excellent	Reddish orange	Yellowish Grey	Strong Brown
Glycerol-aspargine agar (ISP 5)	Good	--	Yellowish White	Yellowish White
Peptone Yeast extract Iron agar (ISP 6)	Good	Light Yellow	Yellowish Pink	Brownish Pink
Tyrosine agar	Good	--	Greenish White	Strong Brown
Bennets agar	Excellent	Reddish Brown	Light Gray	Dark Red
Streptomyces agar	Excellent	Reddish Brown	Light Gray	Dark Red
Starch Casein agar	Good	Light Yellow	Purplish White	Orange Yellow

Supplementary Table S2 : Properties of *Streptomyces sp* OA293

**Characteristic**	***Streptomyces sp* OA 293**
Morphology (In ISP2)	
Spore chain morphology	Rectusflexibilis
Spore surface ornamentation	Smooth
Substrate mycelium	Blackish Red
Growth characteristics on	
Temperature (°C)	15°C-37°C
0-4% NaCl	+
5% NaCl	-
7% NaCl	-
Growth at pH	
8	+
9 & 10	+
Degradation of	
Casein	-
Gelatin	-
DNA	-
Starch	+
Biochemical	
Catalase	+
Nitrate reduction	+
Methyl Red	+
Voges Proskauer	-
Indole	-
Citrate Utilization	+
Carbon Utilization	
L-Arabinose	+
D-Xylose	+
Inositol	-
D-Mannitol	+
D-Fructose	+
L-Rhamnose	+
Raffinose	+
Salicin	+
D- Galactose	+
D-Mannose	+
Maltose	+

Supplementary Figure 1: Full length blots from main text

Fig 3c

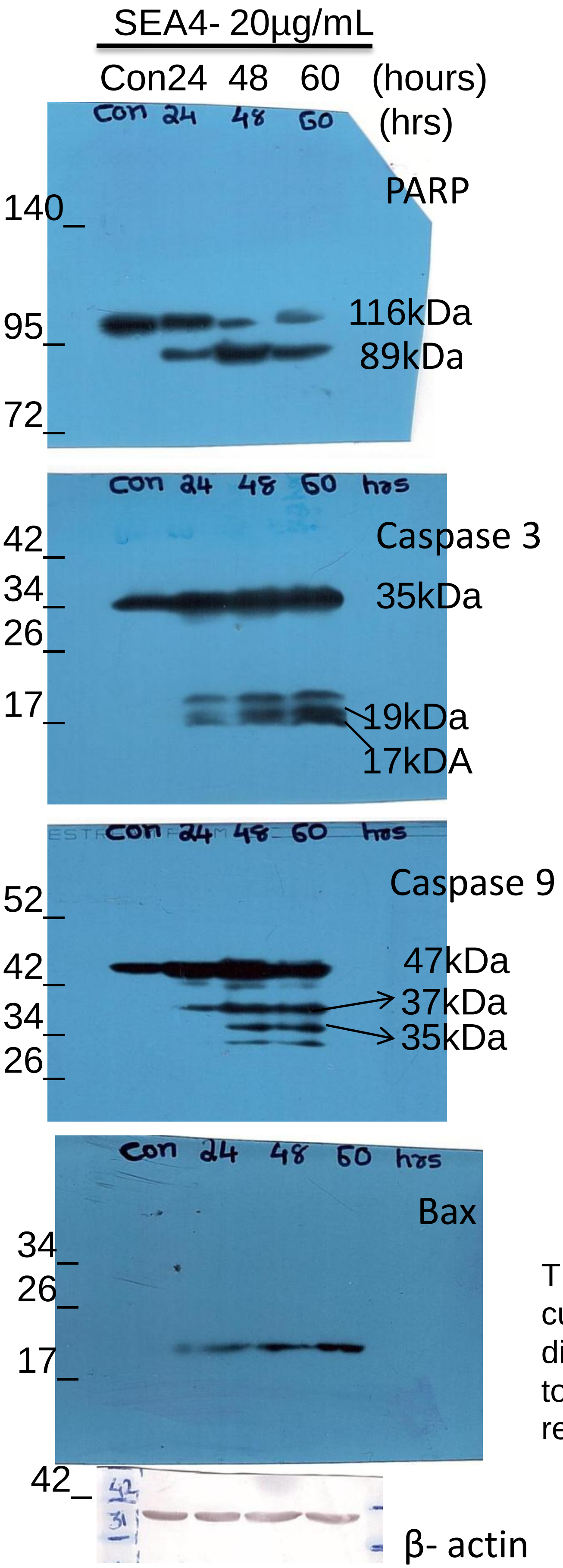

The membranes were cut to probe for different antibodies and to avoid washing and reprobing

Figure 3d

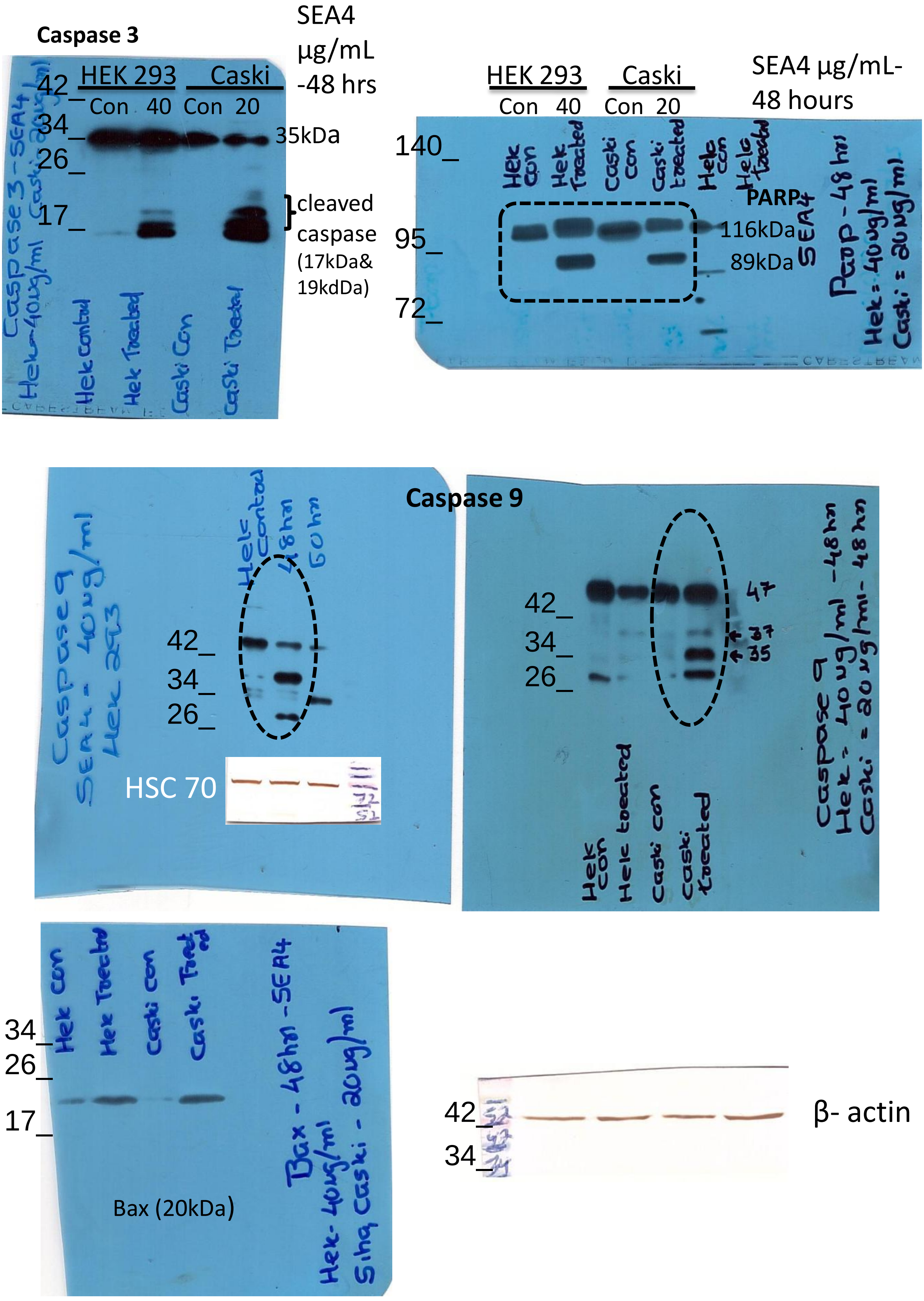
Caspase 3
SEA4 µg/mL -48 hrs
HEK 293
Caski
Con 40 Con 20
42
34
26
17
35kDa
cleaved caspase (17kDa& 19kDa)
HEK 293
Caski
Con 40 Con 20
SEA4 µg/mL- 48 hours
140
95
72
PARP
116kDa
89kDa
Caspase 9
42
34
26
HSC 70
42
34
26
47
37
35
34
26
17
Bax (20kDa)
42
34
β- actin

Figure 5a

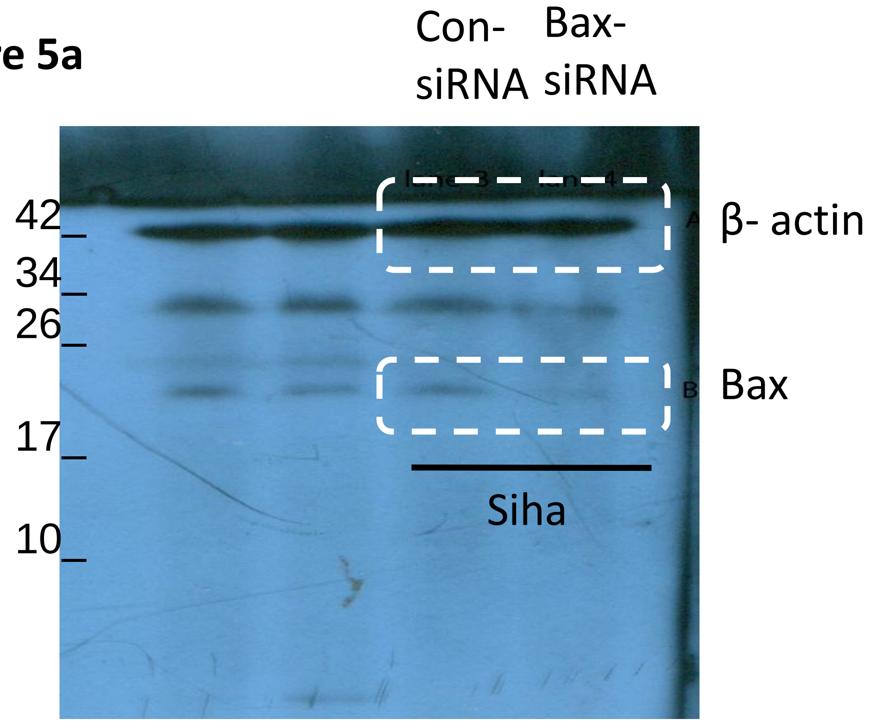

Figure 5c

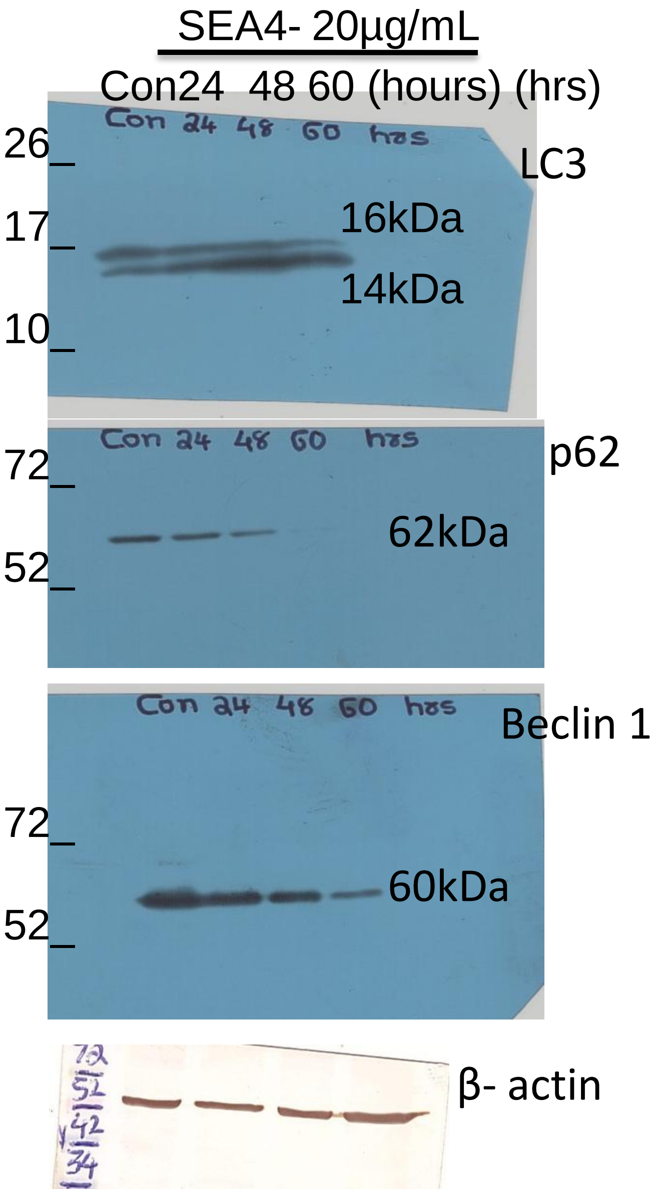

Figure 5d

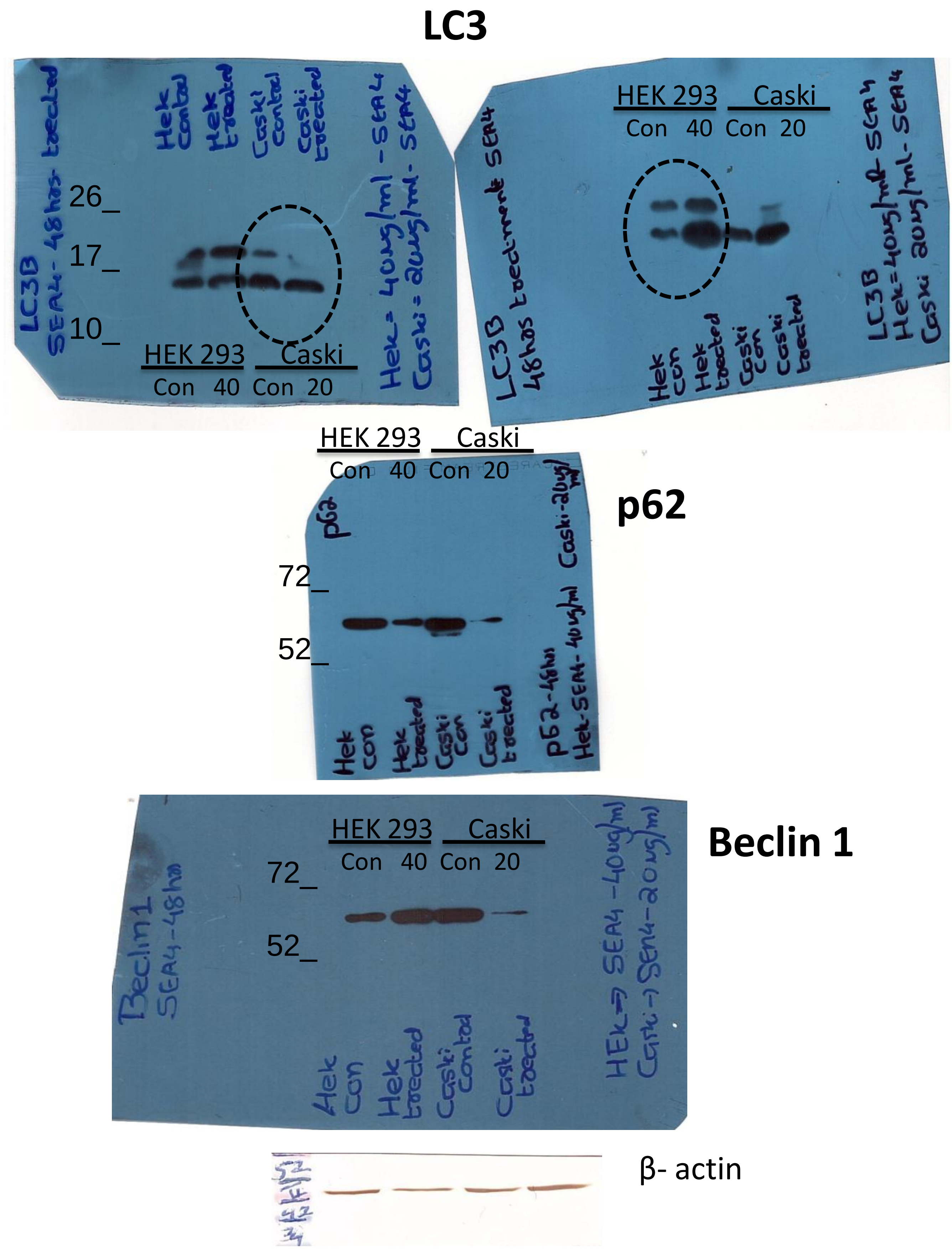

LC3
26_
17_
10_
HEK 293
Caski
Con 40 Con 20
HEK 293
Caski
Con 40 Con 20
HEK 293
Caski
Con 40 Con 20
p62
72_
52_
HEK 293
Caski
Con 40 Con 20
Beclin 1
72_
52_
β- actin

Fig 6a

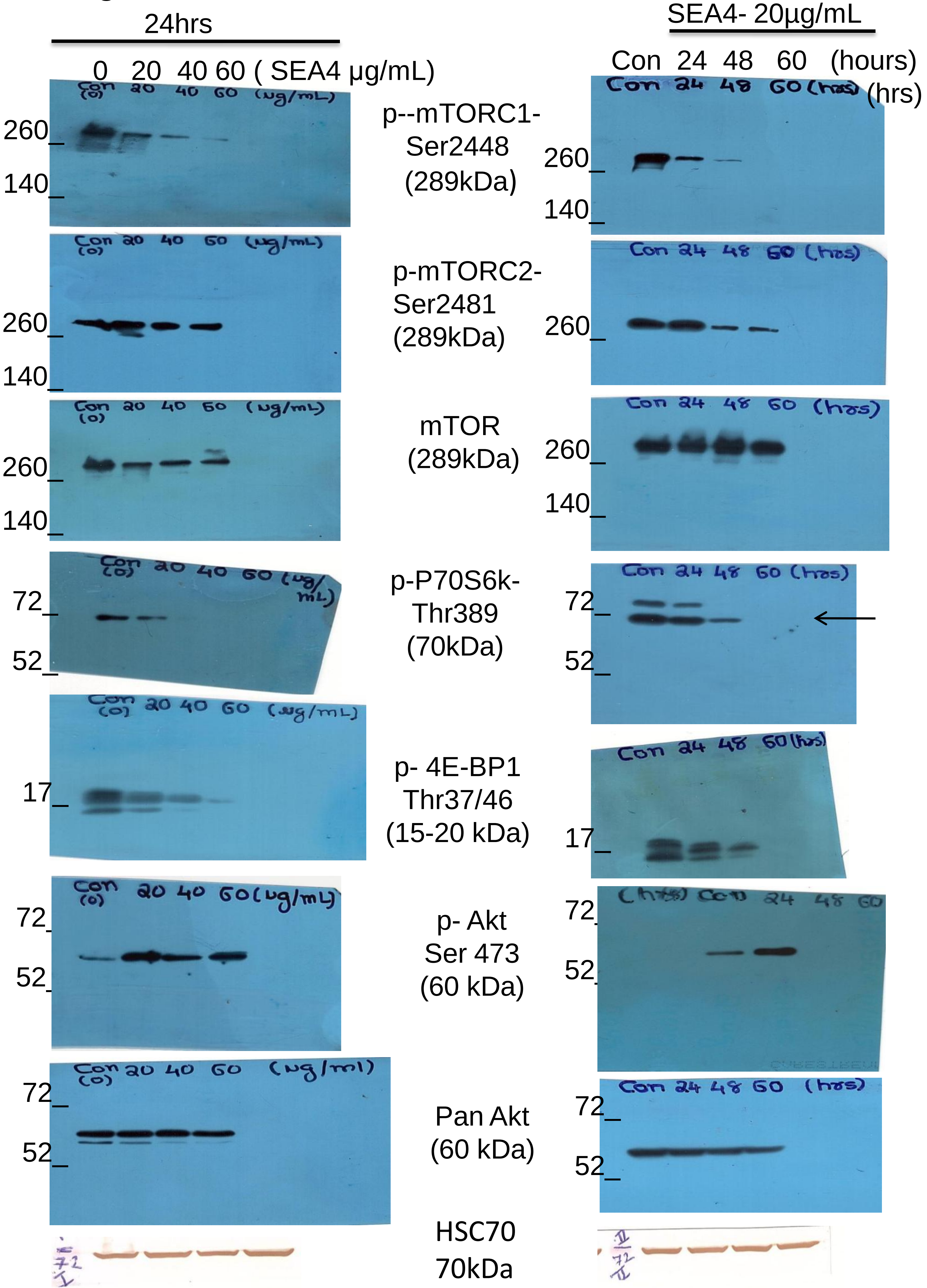
24hrs
0 20 40 60 (SEA4 µg/mL)
SEA4- 20µg/mL
Con 24 48 60 (hours)
(hrs)
p--mTORC1-
Ser2448
(289kDa)
p-mTORC2-
Ser2481
(289kDa)
mTOR
(289kDa)
p-P70S6k-
Thr389
(70kDa)
p- 4E-BP1
Thr37/46
(15-20 kDa)
p- Akt
Ser 473
(60 kDa)
Pan Akt
(60 kDa)
HSC70
70kDa
260
140
72
52
17
